# Perioperative Drug Management of Systemic Therapies in Breast Cancer: A Literature Review and Treatment Recommendations

**DOI:** 10.3390/curroncol32030154

**Published:** 2025-03-09

**Authors:** Mariem Galuia, Julia Fedorova, Wassim McHayleh, Eleftherios Mamounas, Sarfraz Ahmad, Sabrina Pavri

**Affiliations:** 1 Department of Internal Medicine, AdventHealth Hospital, Orlando, FL 32804, USA; mariem.galuia.md@adventhealth.com; 2 College of Medicine, University of Central Florida, Orlando, FL 32827, USA; julia.fedorova@ucf.edu; 3 Department of Medical Oncology, AdventHealth Hospital, Altamonte Springs, FL 32701, USA; 4 Division of Breast Surgery, Department of Surgery, AdventHealth Hospital, Orlando, FL 32804, USA; terry.mamounas.md@adventhealth.com; 5 Gynecologic Oncology Program, AdventHealth Cancer Institute, Orlando, FL 32804, USA; 6 Division of Plastic Surgery, Department of Surgery, AdventHealth Hospital, Orlando, FL 32804, USA; sabrina.pavri.md@adventhealth.com

**Keywords:** breast cancer, perioperative, chemotherapy, immunotherapy, endocrine therapy, targeted therapy: guideline, management, surgery, breast reconstruction, mastectomy, wound healing

## Abstract

Breast cancer accounts for about 30% of all new female cancers each year, and its incidence is increasing 0.6% per year. An enhanced understanding of the molecular mechanisms of carcinogenesis has led to the development of constantly evolving strategies for local and systemic therapies. Perioperative chemotherapy, immunotherapy, and endocrine therapy play pivotal roles in the overall treatment plan. Guidelines on the appropriate use of these drugs in patients undergoing extirpative breast surgery and/or breast reconstruction are lacking. Clear indications for the management of systemic therapies relative to the timing of surgery is crucial to ensure consistent treatment outcomes and to minimize complications. Our purpose is to propose evidence-based recommendations to optimize the perioperative management of systemic therapies in patients undergoing breast cancer surgery and breast reconstructive surgery. In this review, we outline the basic tenets of breast cancer therapies, provide an overview on wound-healing principles, delineate relevant pharmacodynamic concepts, summarize literature and pharmacologic data from various preclinical studies and clinical trials, and propose treatment recommendations. Synopsis: This review proposes evidence-based recommendations regarding systemic therapies management for outcome optimization in the perioperative period in breast cancer patients.

## 1. Introduction/Background

Breast cancer (BC) represents a significant public health concern, with the American Cancer Society estimating that in 2024, 310,720 new cases of invasive breast cancer will be diagnosed, and about 42,250 women will die from breast cancer [1]. Its treatment typically involves a multidisciplinary approach incorporating various therapeutic modalities such as surgery, systemic therapies, and radiation therapy. Neoadjuvant chemotherapy results in equivalent outcomes to adjuvant chemotherapy in patients with operable/locally advanced breast cancer and has emerged as a standard practice for most patients with triple negative breast cancer (TNBC), human epidermal growth factor receptor 2 (HER-2) positive breast cancer, and in select patients with the hormone-receptor positive (HR+)/HER2 negative (HER2-) phenotype. Potential clinical advantages of neoadjuvant chemotherapy include its ability to down-stage the primary breast tumor and involved axillary lymph nodes, leading to a decrease in the extent of the surgical resection in the breast and axilla, as well as the tailoring of adjuvant systemic therapy and adjuvant radiotherapy after surgery [2].

Traditionally, cytotoxic chemotherapy has been a cornerstone of adjuvant therapy in breast cancer management, and patients with HR+ disease also benefit from endocrine therapy. However, recent advancements in understanding the molecular mechanisms of carcinogenesis have led to the development of novel drugs that have dramatically changed patient prognosis. Agents targeting specific receptors that interrupt cellular transduction pathways, immune checkpoint inhibitors (ICIs), poly ADP ribose polymerase (PARP) inhibitors, and antibody–drug conjugates (ADCs) have now become standard clinical practice in breast cancer management. Patients with HER2-positive breast cancer are given HER2-targeted therapies in combination with a chemotherapy backbone, select patients with TNBC are treated with immunotherapy in combination with chemotherapy, high-risk HR+/HER2-negative patients receive endocrine therapy with cyclin-dependent kinase (CDK) inhibitors, and high-risk patients with pathogenic breast cancer (*BRCA*) gene mutations benefit from the use of adjuvant PAPR inhibitors [3].

The administration of systemic therapies around the time of surgical intervention has raised concerns among breast surgeons and reconstructive plastic surgeons regarding the potential for perioperative surgical complications: specifically wound dehiscence, delayed wound healing, and wound infection.

Common cytotoxic chemotherapies used in breast cancer treatment, such as cisplatin, exert their effects by blocking the cell cycle through DNA nucleotide alkylation, which can impede wound healing in the perioperative period. Experimental animal models have shown that preoperative platins inhibit neovascularization and reduce fibroblast proliferation, suggesting potential adverse effects on surgical outcomes [4]. Similarly, doxorubicin has been shown to be cytotoxic to fibroblasts and leukocytes [5]. Animal studies have highlighted its detrimental effect on delayed wound healing, further implicating its potential impact on surgical outcomes when used perioperatively [6]. Additionally, myelosuppression (precisely neutropenia) is a common adverse effect of many chemotherapies and poses an increased risk for wound infection, which is one of the most frequent causes of postoperative morbidity.

A study of the timing of surgery in patients receiving neoadjuvant chemotherapy for breast cancer at MD Anderson Cancer Center demonstrated that preoperative chemotherapy did not increase postoperative morbidity compared to patients who did not receive it [7]. The average time from the completion of chemotherapy to surgery was 27 days (range 6–108), which was determined by the operating surgeon. “Strict operative criteria were used to plan the timing of surgery”, although these criteria were not defined, and none of the patients underwent reconstruction, which somewhat limits the applicability of the conclusions. A limited number of further studies have reported similar results [8,9,10].

It is generally accepted that due to the adverse wound-healing effects, surgery should be delayed for a minimum of 4 weeks after the completion of cytotoxic chemotherapy, and the patient should demonstrate the resolution of any chemotherapy-induced neutropenia. The development and utilization of immunotherapy and targeted therapies in breast cancer has surged in recent years, leading to an increasing number of surgical patients being treated with these novel agents. While demonstrating effective anti-cancer activity, these medications can precipitate a spectrum of immune-related adverse events, including cutaneous manifestations that may impede wound healing when administered in the perioperative period.

To our knowledge, and following a comprehensive literature review, there are no universally accepted guidelines among surgeons and medical oncologists for the perioperative management of systemic therapies in patients undergoing extirpative breast surgery and/or breast reconstruction. Uncertainty persists on whether to continue or suspend specific medications to mitigate the burden of postoperative morbidity, and this is often dealt with on a case-by-case basis by the patient’s surgeon and their medical oncologist team. An extensive review of the literature and pharmacologic data from various preclinical studies and clinical trials was performed. Herein, our objective is to propose evidence-based recommendations to optimize the perioperative management of systemic therapies in patients undergoing breast cancer surgery.

## 2. Methods

The U.S. Food and Drug Administration (FDA)-approved drug label was reviewed and referenced for each drug. Information regarding pharmacodynamics (PD) and pharmacokinetics (PK) including respective half-lives, dosing intervals, and steady states was gathered and considered in our review process. An electronic search of the PubMed and Google database was performed to obtain key peer-reviewed literature. We identified relevant publications by reviewing citations. The specific drug name was researched in the database along with the following keywords: “perioperative”, “wound healing”, “thrombosis”, “bleeding”, “complications”, “infection”, and “breast”. We also considered isolated prospective and retrospective cohorts, proceedings from Scientific Societies’ annual meetings, and ultimately individual case reports when more rigorous data were limited or non-existent. We also cited correspondence with the pharmacological companies of several drugs for which minimal or no data were available.

We reviewed the evidence regarding the impact of the use of chemotherapy, endocrine therapy, immunotherapy, and targeted therapy in the perioperative setting on wound complications, specifically delayed wound healing, wound dehiscence, and infection. Other factors considered included bleeding risk, microvascular flap complications, and thromboembolic events such as deep vein thrombosis (DVT) and pulmonary embolus (PE).

An understanding of the basic principles of pharmacokinetics, and most importantly the concept of half-life, is necessary to determine useful parameters such as the dosing interval, the duration of action of a drug, the time required to attain a steady-state plasma concentration within a multi-drug regimen, as well as the elimination rate. The elimination half-life of a drug is a function of both clearance (Cl) and volume distribution (Vd) and is considered to be independent of the drug concentration. This implies that pathophysiologic changes that result in changes in Vd and Cl can affect drug elimination. We also recognize that to utilize half-life as a basis for our recommendations, we are assuming elimination by first-order kinetics and a close relationship of drug effect to its plasma concentration. We are aware of the limitations of this assumption as these criteria may not be applicable to all the drugs listed. At any given dose, it takes 4 to 5 half-lives to eliminate 97% of a drug from the body [11]. A thorough review of the elimination half-lives of each specific drug used perioperatively in breast cancer therapy was conducted and guided our recommendations when relevant preclinical data were not available (Table 1, Table 2, Table 3 and Table 4).

Following an extensive literature review, evidence-based data were presented and carefully examined in regular multidisciplinary meetings that brought together specialists from breast oncology, breast surgery, and plastic surgery. In this collaborative approach, each expert contributed their unique perspectives, ensuring that all aspects of the subject were considered, from the specificities of therapies to details of surgical techniques. Our research proposes well-informed recommendations based on a thorough examination of existing research studies, evaluation of existing evidence and expert consultation.

## 3. Findings

### 3.1. Chemotherapy

Chemotherapy agents include anthracyclines, taxanes, microtubule dynamics inhibitors, platinum agents, and antimetabolites. Generally, patients undergoing chemotherapy treatment should not undergo elective surgery within 4 weeks of being on chemotherapy due to diminished capacity for wound healing. Additionally, white blood cell counts (most importantly absolute neutrophil counts) as well as platelet counts should be monitored regularly to mitigate the risk of wound infection and/or bleeding. If these agents need to be restarted in the postoperative period, we recommend that they not be resumed until at least 4 weeks postoperatively, assuming there is no evidence of wound-healing problems, surgical site infection, or systemic infection (Table 1).

*Anthracyclines:* Doxorubicin (Adriamycin) and Epirubicin (Ellence) are anthracyclines indicated for the treatment of early-stage and advanced breast cancer. Anthracyclines intercalate into DNA, causing the breakage of DNA strands and inhibition of DNA synthesis. Additionally, they disrupt topoisomerase II, a critical enzyme for DNA unwinding for replication and synthesis, thereby causing growth arrest and programmed cell death [12]. Doxorubicin (Adriamycin) has the added property of generating free radicals and increasing oxidative stress. Animal studies have shown that anthracyclines can impede healing by decreasing wound cellularity and collagen synthesis [5,6]. Devereux et al. reported a decrease in collagen fiber diameter and lower hydroxyproline levels on days 14 and 21 days after administration in rats [13]. De Cunzo et al. reported that doxorubicin can cause decreased wound tensile strength [14].

*Taxanes:* Taxanes are indicated for breast cancer treatment in the early-stage and advanced disease setting and include Paclitaxel (Taxol), Docetaxel (Taxotere), and Albumin-bound paclitaxel (Abraxane). These drugs block cell cycle progression through centrosomal impairment, induction of abnormal spindles and the suppression of normal spindle microtubule dynamics. Paclitaxel binds to tubulin protein subunits in microtubules, which stabilizes them and disrupts the dynamic equilibrium of microtubule assembly and disassembly. This process arrests cells in the G2/M phase of the cell cycle, causing mitotic arrest and cell death [15]. Docetaxel inhibits physiological microtubule depolymerization and disassembly by binding to and stabilizing tubulin, thus causing cell cycle arrest in the G1/M phase, eventually leading to cell death [16]. The absolute neutrophil count (ANC) should be monitored to mitigate infection risk while using this drug class.

*Platinum Agents:* Platinum agents are used usually in combination for treatment of early-stage, locally advanced or metastatic breast cancer. Carboplatin (Paraplatin) is an atypical alkylator that forms interstrand DNA crosslinks, inhibiting DNA repair, and leading to DNA strand breakage during replication [17]. Animal studies have shown that platinum agents can cause a dose-dependent decrease in wound tensile strength by reducing fibroblast proliferation [18].

*Antimetabolites:* Antimetabolites are indicated mostly in metastatic breast cancer or in the post-neoadjuvant chemotherapy setting and include the drugs Capecitabine (Xeloda) and Gemcitabine (Gemzar). Capecitabine is a 5-fluorouracil (5-FU) prodrug that interferes with DNA synthesis by blocking progression from the G_1_ to the S phase. This drug inhibits thymidylate synthetase, blocking the methylation of deoxyuridylic acid to thymidylic acid. Capecitabine can be used either in combination or as a monotherapy. Gemcitabine blocks the progression of cells through the G_1_ to S phase boundary by inhibiting ribonucleotide reductase [19]. Gemcitabine is typically used in the advanced disease setting after the failure of anthracycline and/or taxanes [20]. While antimetabolites are not associated with wound complications in experimental studies, delayed wound healing has been reported in vivo [21,22,23].

### 3.2. Endocrine Therapy

Endocrine therapy agents used in the treatment of breast cancer include SERMs (selective estrogen receptor modulators), SERDs (selective estrogen receptor degraders), and SAIs (selective aromatase inhibitors). Endocrine therapy is used primarily for patients with ER+ cancers in the adjuvant as well as in the advanced disease setting (Table 2).

*SERMs:* Tamoxifen is an SERM, which is indicated in ER+ breast cancer. Tamoxifen competes with 17β-estradiol (E_2_) at the receptor site and blocks the promotional role of E_2_ in the breast tissue, which can result in a decrease in estrogen receptor signaling-dependent growth in breast tissue [24]. Tamoxifen has a theoretical association with poor wound healing as it decreases transforming growth factor (TGF)-β2, thereby reducing the production of collagen—it is unclear as to whether this is clinically significant. Initial preclinical data did not comment specifically on wound-healing risks but did note an increased risk for thromboembolic events. Data from the NSABP P-1 trial demonstrated a statistically significant increase in the risk of PE and stroke and a non-statistically significant increase in the risk of DVT. Additionally, a few retrospective studies of breast cancer patients undergoing surgery while on tamoxifen seem to suggest an increased risk of delayed wound healing, fat necrosis, infections, and grade III/IV capsular contracture [25]. While robust prospective data are lacking, based on the mechanism of action and existing clinical evidence, it is reasonable to recommend holding tamoxifen for 1 week before and 3 weeks after the surgery.

*SERDs:* Fluvestant and Elasestrant possess exclusive antagonist effects, which competitively bind, block, and accelerate the degradation of estrogen receptors. Fluvestant is indicated for HR+ and HER2- advanced breast cancer in postmenopausal women [26]. Elacestrant is indicated for postmenopausal women or adult men with ER+, HER2-, estrogen receptor 1 (ESR1)-mutated advanced or metastatic breast cancer with disease progression after prior endocrine therapy [27]. During the phase III EMERALD study, one of the exclusion criteria was major surgery less than 28 days before the first dose of the study drug. Elacestrant’s effect on wound healing was evaluated in rats who received oral Elacestrant [28]. There were no significant differences between Elacestrant treatment and control animals in wound healing at designated time points up to Day 29. Therefore, based on exclusion criteria from preclinical trial data, we recommend withholding SERDs for 4 weeks prior and after the surgery.

*Selective Aromatase Inhibitors:* Letrozole Anastrozole and Exemestane are selective aromatase inhibitors (AI) indicated as adjuvant therapy for postmenopausal women with early-stage, node-positive, and ER+ BC as well as in the advanced disease setting. Selective aromatase inhibitors block the conversion of androgens to estrogens in the adrenal glands, skin, muscle, and adipose tissue [29,30,31]. AIs appear to be associated with a higher risk of postoperative wound healing complications and capsular contracture [25]. Similar to tamoxifen, withholding aromatase inhibitors in the perioperative period may help decrease the risk of wound-healing complications, especially in the case of prosthetic reconstruction. We recommend holding aromatase inhibitors 1 week prior to surgery and resuming 3 weeks postoperatively.

### 3.3. Immunotherapy

Immunotherapy enhances a patient’s native immune system, increasing its efficacy in recognizing and destroying neoplastic cells. Typically, these therapies work on specific proteins to boost the immune response. Currently, there is one drug class of immunotherapy (immune checkpoint inhibitors) that is used to treat breast cancer. Here, we summarize proposed treatment recommendations for specific immunotherapies perioperatively (Table 3).

*Immune Checkpoint Inhibitors:* Pembrolizumab (Keytruda) is an immune checkpoint inhibitor, affecting PD-1 (programmed cell death protein 1). It is a monoclonal antibody (Ab) that is directed against PD-1 on lymphocyte T-cell surfaces. This blocks the interaction with the PD-1 ligands, programmed death-ligand 1 (PD-L1) and PD-L2. The binding of PD-1 to these ligands inhibits T-cell activity against neoplastic cells [32]. Pembrolizumab is indicated for high-risk early stage, metastatic, or recurrent TNBC [33]. While initial preclinical trials were designed to hold pembrolizumab 3–5 weeks prior to surgery [34,35,36], subsequent clinical studies have found no evidence that wound healing is affected by continuing pembrolizumab in the perioperative period [37]. Therefore, it seems reasonable to continue using pembrolizumab in the perioperative setting.

*Antibody-Drug Conjugates:* Antibody–drug conjugates used in the treatment of breast cancer include Ado-trastuzumab emtansine Fam-trastuzumab deruxtecan and Sacituzumab govitecan. Ado-trastuzumab emtansine (or T-DM1) is a trastuzumab-microtubule inhibitor conjugate, which causes cell cycle arrest and apoptotic cell death. The ADC binds to HER2 and is subsequently internalized and degraded, resulting in the release of an active metabolite (lysine-MCC-DM1). The active metabolite can bind with tubulin, disrupting microtubule networks in the cell. This medication is indicated for the treatment of HER2+ BC with residual disease after neoadjuvant chemotherapy plus HER2 targeted therapy [38,39]. We recommend holding ado-trastuzumab for 4-weeks preoperatively and for 3–4 weeks postoperatively.

Fam-trastuzumab deruxtecan is an ADC specifically targeting HER2+ breast cancer. This medication is a trastuzumab–topoisomerase inhibitor conjugate, which binds to HER2 and undergoes internalization and cleavage by lysosomal enzymes. This cleaved metabolite then enters the nucleus, causing DNA damage and apoptosis [40]. While there are no recommendations in the fam-trastuzumab deruxtecan prescribing information for use in patients undergoing surgery or who have recently received surgery, patients who underwent major surgery within 4 weeks prior to randomization were excluded from the clinical trials [41,42,43,44]. Based on this exclusion criteria, we recommend against using fam-trastuzumab deruxtecan within 4 weeks of surgery.

Sacituzumab govitecan (Trodelvy) is a Trop-2-directed antibody and topoisomerase inhibitor conjugate drug that targets the Trop-2 antigen, expressed on various tumor cells, and delivers 7-ethyl-10-hydroxycamptothecin (SN-38; a topoisomerase I inhibitor), which causes single strand breaks, inducing DNA damage and apoptosis. This medication is indicated for locally advanced and metastatic BC [45]. Most importantly, patients who underwent major surgery within 4 weeks were excluded from the phase III ASCENT trial [46]. No wound complications were reported. We recommend withholding sacituzumab govitecan for 4 weeks prior to and after surgery, and patients should be monitored for neutropenia prior to surgery (Table 3).

At the present time, there is a lack of evidence-based data regarding the effect on wound healing of these antibody–drug conjugates, but due to exclusion criteria in the clinical trials, it is recommended to hold these medications for 4 weeks prior to surgery. There are also no peer-reviewed published recommendations as to when to restart them; however, due to the fact that the HER2 and Trop-2 antigens are expressed on a variety of cell types (including a variety of cell types in the skin), it stands to reason that holding the drug for 3–4 weeks after surgery (or until healing is confirmed) is a reasonable (albeit conservative) course of action.

### 3.4. Targeted Therapy

Targeted therapy is an expansive category that encompasses anti HER2 monoclonal antibodies, anti-HER2 tyrosine kinase inhibitors, cyclin-dependent kinase 4/6 (CDK4/6) inhibitors, phosphatidylinositol 3-kinase (PI3K) inhibitors, PARP inhibitors, and protein B kinase (AKT) inhibitors. These therapies work by targeting proteins found on cancer cells to either destroy tumor cells or inhibit cell growth. Below and in Table 4, we summarize the proposed key treatment recommendations for specific targeted therapies perioperatively.

*Anti-HER2 Monoclonal Antibodies:* Anti HER2 monoclonal antibodies are a class that includes Trastuzumab, Pertuzumab, and Trastuzumab/Pertuzumab/hyaluronidase injection). These therapies are HER2/neu receptor antagonists. Trastuzumab is a monoclonal antibody that binds to the HER2 receptor to block the cleavage of its extracellular domain to induce down-modulation. This mediates antibody-dependent cellular cytotoxicity (ADCC) and leads to cell cycle arrest and the suppression of cell growth and proliferation. Trastuzumab in combination with chemotherapy is indicated as adjuvant/neoadjuvant therapy for early-stage/locally advanced breast cancer and as first-line treatment for HER2+ metastatic BC [47]. Pertuzumab acts as an antagonist by inhibiting HER2 dimerization and activation. It is indicated for the treatment of metastatic HER2+ BC in individuals who have not received prior therapy as well as for the neoadjuvant treatment of HER2+ early-stage/locally advanced BC and adjuvant treatment for HER2+ early-stage BC at high risk of recurrence [48]. Trastuzumab/Pertuzumab/hyaluronidase injection is a subcutaneously administered combination therapy of pertuzumab and trastuzumab, which is indicated for the neoadjuvant treatment of HER2+, locally advanced, inflammatory, or early-stage BC, and adjuvant treatment of HER2+ early BC with an elevated risk of recurrence [49].

Trastuzumab alone is not associated with surgical site complications. However, combination therapy with pertuzumab has been associated with an increased risk of wound breakdown after immediate breast reconstruction, requiring additional surgical intervention. This effect may be due to the activation of complement and local inflammatory reaction, which is detrimental in terms of wound repair [50,51,52]. We recommend proceeding with surgical intervention if the patient is receiving trastuzumab alone. If the patient is receiving pertuzumab or combination targeted HER2 treatment, we recommend withholding the therapy 4 weeks before surgery.

*Anti-HER2 Tyrosine Kinase Inhibitors:* Lapatinib Neratinib, and Tucatinib are anti-HER2 tyrosine kinase inhibitors (TKIs). They bind to the tyrosine kinase domain in the HER2 protein, blocking the activation of the signaling pathway and resulting in cell cycle arrest and apoptosis. All three therapies are indicated in advanced or metastatic treatment of HER2+ BC with a history of prior therapy [53,54]. Neratinib is also indicated as an extended adjuvant treatment of adult patients with early stage HER2 over-expressed/amplified breast cancer to follow adjuvant trastuzumab-based therapy.

Lapatinib is a 4-anilinoquinazoline kinase inhibitor of the intracellular tyrosine kinase domains of both epidermal growth factor receptor (EGFR [ErbB1]) and of HER2 [ErbB2], which inhibits ErbB-driven tumor cell growth. Lapatinib has demonstrated an anti-proliferative effect on HER2+ tumors with increased epiregulin levels (epiregulin being a ligand for EGFR and HER4) as well as a subset of HER2- tumors with increased HER3 mRNA expression [55]. Multiple studies investigating the efficacy of lapatinib in combination with trastuzumab in the neoadjuvant setting for treatment of HER2+ breast cancer have not noted any adverse wound-healing side effects after surgery even with continuing both therapies up until the day of surgery [56]. Therefore, we recommend that lapatinib is continued through the perioperative period (Table 4).

Neratinib is a TKI that binds to the intracellular domain of HER1, HER2, and HER4 receptors, inhibiting downstream cell signaling pathways and reducing tumor growth and proliferation. It is not known to interfere with the vascular endothelial growth factor (VEGF) signaling pathways like other VEGF-tyrosine kinase inhibitors that are associated with impaired wound healing and angiogenesis [57]. Post-marketing safety analyses have not reported any safety signals or increased incidence of impaired wound healing among patients treated with Neratinib [58]. We recommend that neratinib therapy is continued through the perioperative period (Table 4).

While there are currently no available data on the effect of tucatinib on wound healing (a clinical trial is ongoing) [59], the phase II study protocol required the discontinuation of tucatinib 3 to 7 days prior to surgery, and treatment was resumed 3 to 21 days postoperatively [60]. We recommend that tucatinib is withheld 3 days prior to surgery and therapy is withheld until at least 3 days postoperatively. Phase III clinical trials excluded patients undergoing surgical intervention [61].

*Cyclin-Dependent Kinase 4/6 Inhibitors:* Palbociclib, Ribociclib, and Abemaciclib are cyclin-dependent kinase 4/6 inhibitors. These medications target CDK4 and CDK6 proteins on cancer cells, inhibiting cellular proliferation and blocking the progression from G1 to S phase in the cell cycle. All three medications are indicated for the treatment of HR+, HER2+ advanced or metastatic BC [62,63]. Abemaciclib is additionally indicated for the adjuvant treatment of HR+, HER2-, node-positive early-stage breast cancer at high risk of recurrence [64].

A search of the peer-reviewed published medical literature failed to identify any references discussing the perioperative use of Palbociclib and associated wound healing issues. As per the PALOMA-1, 2 and 3 study protocols, caution was advised on theoretical grounds for any surgical procedures during the study. Based on the available pharmacokinetic data, stopping palbociclib was recommended at least 7 days prior to surgery. Postoperatively, the decision to reinitiate palbociclib treatment was to be based on a clinical assessment of satisfactory wound healing and recovery from surgery. It seems reasonable to restart at 3 weeks postoperatively given the surgical wound is healed (Table 4).

The Ribociclib prescribing information does not reference impaired wound healing or wound-related complications as an adverse event (AE). However, grade 1 and grade 2 wound-related adverse events were reported across all three pivotal clinical studies of Ribociclib, and neutropenia is a common side effect of this drug [65,66,67]. Neutropenia associated with CDK4/6 inhibitors is different from chemotherapy-induced neutropenia in that it is rapidly reversible due to a cytostatic (as opposed to cytotoxic) effect on neutrophil precursors in the bone marrow. Similar to Palbociclib, it is recommended to stop Ribociclib 7 days prior to surgery and restart at 3 weeks postoperatively as long as wound healing is confirmed [68].

Abemaciclib is structurally distinct from palbociclib and ribociclib, with greater CDK4 selectivity, and less risk of hematologic adverse effects such as neutropenia. Information on holding abemaciclib treatment for surgery is available in several clinical study protocols, and based on these, we recommend that abemaciclib is discontinued at least 7 days prior to surgery and is held at least 14 days postoperatively [69,70,71].

*mTOR Inhibitors:* Everolimus inhibits mammalian target of rapamycin (mTOR), a serine threonine kinase, by binding to an intracellular protein (FKBP-12) and mTOR complex 1, resulting in inhibition of mTOR kinase activity. It also inhibits the expression of hypoxia inducible factor (HIF-1) and reduces the expression of VEGF, reducing cell proliferation, angiogenesis, and glucose uptake in in vitro and in vivo studies. Everolimus is indicated for postmenopausal women with advanced HR+ and HER2- BC in combination with exemestane [72]. The prescribing information does reference the risk of impaired wound healing as a warning and precaution and recommends that everolimus is held 1 week prior to surgery and restarted 2 weeks after surgery as long as the wound is healing. In several clinical trials conducted with everolimus in patients with advanced HR+, HER2- BC, study drugs were interrupted one week prior to surgery and restarted as soon as possible after wound healing [73,74,75]. Phase III clinical trials did report minor grade 1/2 wound-healing side effects; however, subsequent clinical data have not demonstrated significant wound-healing side effects. In a study involving everolimus administration up until the day prior to surgical resection for operable non-small cell lung cancer, none of the 23 patients in the treatment arm were reported to have any post-surgical wound-healing issues. We currently recommend that everolimus is withheld for at least one week prior to surgery and not restarted until 2 weeks or until adequate wound healing is achieved. However, we recognize that if further clinical data reinforce the above findings, the recommendation may be changed in the future to continue everolimus through the perioperative period (Table 4).

*PI3K Inhibitors:* Alpelisib is a PI3K inhibitor indicated for use (typically in combination with fulvestrant) in postmenopausal women and men with HR+ HER2-negative, PIK3CA-mutated advanced or metastatic BC [76]. This medication targets breast cancer cells with the PIK3CA gene mutation, inhibiting the PI3Kα and Akt-signaling pathway and subsequent tumor growth. In the phase III SOLAR-1 trial, patients who had surgery within 14 days prior to starting the study drug or who had not recovered from major side effects were excluded [77,78]. Due to the possibility of severe hyperglycemia associated with alpelisib use (which can be upwards of 60%), glucose levels should be monitored closely in diabetic patients in the perioperative period, and tight glycemic control should be prioritized to minimize the adverse effect of hyperglycemia on wound healing and the risk for surgical site infection [79]. A comprehensive search of the peer-reviewed literature did not identify any complication associated with the use of alpelisib in relation to surgery. As alpelisib specifically targets PIK3CA mutated cells, we recommend that this therapy is continued through the perioperative period (Table 4).

*PARP Inhibitors:* Olaparib and Talazoparib are PARP inhibitors indicated for deleterious germline BRCA mutations and HER2 negative metastatic BC. Both medications inhibit PARP, thus blocking the repair of single-strand DNA breaks. This results in a synthetic lethality of BRCA-associated cancer cells [80,81]. The PARP inhibitors have been implicated in combating fibrosis; however, extensive research has not been carried out regarding their effect on wound healing.

While there are currently no available data on the effect of olaparib on surgical wound healing, the phase II and phase III study protocols required the discontinuation of olaparib 3 days prior to surgery with reinitiation of treatment approximately 10 days afterward. An additional 7 days was permitted if the wound was insufficiently healed, and the patient could recommence olaparib if there was no evidence of disease progression [82,83,84,85,86]. We recommend that olaparib is withheld 3 days prior to surgery and restarted at two weeks provided the wound is healing normally (Table 4). This recommendation may change in the future, as murine models suggest it could actually accelerate wound healing [87], and a study on its use for gynecological malignancies in the neoadjuvant setting is ongoing and will likely report perioperative safety data [88]. The effects on wound healing of Talazoparib have also not been studied in the perioperative setting, but due to a similar mechanism of action, it is reasonable to conclude that the holding parameters should be similar to Olaparib (Table 4).

*AKT Inhibitors:* Capivasertib is a serine and threonine kinase AKT inhibitor as well as an inhibitor of phosphorylation of downstream AKT substrates. Capivasertib is indicated in combination with fulvestrant for HR+, HER2-, locally advanced or metastatic breast cancer with one or more PIK3CA/AKT1/PTEN alterations [89]. The phase III clinical trial CAPItello-291 excluded patients who underwent major surgery within 4 weeks prior to study treatment initiation [90]. The prescribing information does reference the risk of impaired wound healing as a warning and precaution. We recommend that Capivasertib is withheld 4 weeks prior to surgery and for 4 weeks postoperatively (Table 4).

## 4. Discussion

After surgical intervention and subsequent tissue injury, the process of wound healing is initiated and typically progresses through four phases: homeostasis, inflammation, proliferation, and maturation [91]. Homeostasis begins within minutes after the tissue injury occurs with the purpose of preventing blood loss. The coagulation cascade is activated, and the aggregation of platelets forms a blood clot, limiting blood flow. Additionally, the blood clot serves as a provisional matrix [92]. Inflammation is initiated immediately after injury occurs and continues for approximately 2–3 days. Immune cells are recruited to clear any debris and pathogens that are present and to release growth factors that stimulate tissue repair. Fibroblasts migrate to the wound site and secrete collagen, adding to the provisional matrix that bridges the wound [93]. Proliferation then takes place from around 3 days to 3 weeks and includes granulation tissue deposition, re-epithelialization, and wound contraction [94]. The release of growth factors induces fibroplasia and angiogenesis, which is necessary for granulation tissue formation and wound closure [95]. Typically, the granulation tissue is formed after 5 days and replaces the fibrin clot formed during homeostasis. Maturation or remodeling begins after 3 weeks and can continue for up to 2 years [96]. The matrix undergoes remodeling by the realignment and crosslinking of collagen fibers, leading to an increase in tensile strength. Maximal strength is reached at 6 weeks; however, the strength of the scar only approaches 80% of the pre-injury strength. Additionally, this timeline depends on many factors such as wound size, location, and the patient’s immune system [97].

We have based our recommendations on when to hold and resume these systemic therapies in the perioperative period on the best presently available data and evidence (Table 1, Table 2, Table 3 and Table 4). We acknowledge that due to the novelty of many of these agents, the exclusion of surgical patients in the initial clinical trials, and the expansion of indications from metastatic to earlier stage patients, many of these recommendations will likely evolve with time.

## 5. Conclusions

Breast cancer management often requires a comprehensive approach, involving collaboration among various medical specialties. Perioperative chemotherapy, immunotherapy, and endocrine therapy play pivotal roles in the overall treatment strategy. Continued research endeavors have led to the refinement of breast cancer management protocols, resulting in an expanding array of therapeutic options. The dearth of data regarding the perioperative effects of newer drugs was anticipated, underscoring the need for post-marketing surveillance to detect unforeseen adverse events in a real-world clinical practice. We acknowledge that these recommendations are subject to change as more data emerge regarding wound-healing complications for patients who undergo surgery while on these recently approved systemic therapies, especially for those that excluded surgical patients in their clinical trials.

In contrast, one might expect extensive clinical evidence for older drugs with established safety profiles to inform clear recommendations regarding the use of these agents, to make definitive recommendations for withholding and recommencing systemic agents in the perioperative setting. Surprisingly, unambiguous evidence regarding the association of conventional drugs with perioperative complications remains limited. Our recommendations stem from a comprehensive literature review and aim to guide perioperative treatment decisions (Table 1, Table 2, Table 3 and Table 4). However, it is imperative for physicians to individualize perioperative treatment plans based on each patient’s unique characteristics, including comorbidities, renal and hepatic function, performance status, and medication interactions. Ultimately, stopping and re-starting these medications should be at the discretion of the surgeon and the patient’s medical oncologist team.

## Figures and Tables

**Table 1:** A summary of the proposed treatment recommendations for specific ***chemotherapies*** perioperatively.

Chemotherapy	Drug Name	Elimination Half-Life	Perioperative Recommendation
Anthracyclines	Doxorubicin (Adriamycin)	20–48 h	For all cytotoxic chemotherapeutic agents, we recommend holding 4 weeks prior to surgery. These agents can be restarted 4 weeks after surgery provided there is no evidence of wound healing problems, surgical site infection, or systemic infection
Taxanes	Paclitaxel (Taxol)	20.2 h	
	Docetaxel (Taxotere)	4 min	
	Albumin-bound paclitaxel (Abraxane)	27 h	
Platinum-based	Carboplatin (Paraplatin)	1.2 h	
Antimetabolites	Capecitabine (Xeloda)	0.75 h	
	Gemcitabine (Gemzar)	1.7–19.4 h	

**Table 2:** A summary of the proposed treatment recommendations for specific ***endocrine therapies*** perioperatively.

Endocrine Therapy	Drug Name	Elimination Half-Life	Perioperative Recommendation
SERM	Tamoxifen (Soltamox)	5–7 days	Hold for 1 week prior to surgery and for 3 weeks postoperatively
SERD	Fluvestant (Faslodex)	45 days	Hold for 4 weeks prior to surgery and for 4 weeks postoperatively
	Elacestrant (Orserdu)	40 h	Hold for 4 weeks prior to surgery and for 4 weeks postoperatively
Selective Aromatase Inhibitors	Letrozole (Femara)	2 days	Hold for 1 week prior to surgery and for 3 weeks postoperatively
	Anastrozole (Arimidex)	50 h	Hold for 1 week prior to surgery and for 3 weeks postoperatively
	Exemestane (Aromasin)	24 h	Hold for 1 week prior to surgery and for 3 weeks postoperatively

*Abbreviations:* SERM, selective estrogen receptor modulator; SERD, selective estrogen receptor degrader.

**Table 3:** A summary of the proposed treatment recommendations for specific ***immunotherapies*** perioperatively.

Immunotherapy	Drug Name	Elimination Half-Life	Perioperative Recommendation
Immune Checkpoint Inhibitors (ICIs)	Pembrolizumab (Keytruda)	26 days	Continue
Antibody–Drug Conjugates (ACDs)	Ado-trastuzumab emtansine (Kadcyla)	4 days	Hold for 4 weeks preoperatively and for 3–4 weeks postoperatively
	Fam-trastuzumab deruxtecan (Enhertu)	5.6 days	Hold for 4 weeks preoperatively and for 3–4 weeks postoperatively
	Sacituzumab govitecan (Trodelvy)	23.4 h	Hold for 4 weeks preoperatively and for 3–4 weeks postoperatively

**Table 4:** A summary of the proposed treatment recommendations for specific ***targeted therapies*** perioperatively.

Targeted Therapy	Drug Name	Elimination Half-Life	Perioperative Recommendation
HER2 Inhibitors	Trastuzumab (Herceptin)	6 days	Continue
	Pertuzumab (Perjeta)	18 days	Hold for 4 weeks prior to surgery and for 4 weeks postoperatively
	Phesgo (Trastazumab, pertuzumab, and hyaluronidase injection)	12 h	Hold for 4 weeks prior to surgery and for 4 weeks postoperatively
Anti-HER2 Tyrosine Kinase	Lapatinib (Tykerb)	24 h	Continue
	Neratinib (Nerlynx)	12 h	Continue
	Tucatinib (Tukysa)	11.9 h	Hold 3 days preoperatively and for 3 days postoperatively
Cyclin-Dependent Kinase 4/6 Inhibitors	Palbociclib (Ibrance)	29 h	Hold 1 week preoperatively, can be restarted at 3 weeks if surgical wound is healed
	Ribociclib (Kisquali)	42 h	Hold 1 week preoperatively, can be restarted at 3 weeks if surgical wound is healed
	Abemaciclib (Verzenio)	18.3 h	Hold 1 week preoperatively, can be restarted at 2 weeks postoperatively
mTOR Inhibitors	Everolimus (Afinitor)	30 h	Hold 1 week preoperatively, can be restarted at 2 weeks if surgical wound is healed
PI3K Inhibitors	Alpelisib (Piqray)	8-9 h	Continue
PARP Inhibitors	Olaparib (Lynparza)	14.9 h	Hold 3 days prior to surgery, can be restarted at 2 weeks if wound is healing
	Talazoparib (Talzenna)	90 h	Hold 3 days prior to surgery, can be restarted at 2 weeks if wound is healing
AKT Inhibitors	Capivatersib (Truqap)	8.3 h	Hold for 4 weeks prior to surgery, and for 4 weeks postoperatively

*Abbreviations:* HER2, human epidermal growth factor receptor 2; mTOR, mammalian target of rapamycin; PI3K, phosphoinositide 3-kinase; PARP, poly (ADP-ribose) polymerase; AKT, protein kinase B.
